# Coxsackievirus A6 Induces Cell Cycle Arrest in G0/G1 Phase for Viral Production

**DOI:** 10.3389/fcimb.2018.00279

**Published:** 2018-08-15

**Authors:** Zengyan Wang, Yue Wang, Shaohua Wang, Xiangling Meng, Fengmei Song, Wenbo Huo, Shuxia Zhang, Junliang Chang, Jingliang Li, Baisong Zheng, Yanqiu Liu, Yahong Zhang, Wenyan Zhang, Jinghua Yu

**Affiliations:** ^1^Department of Internal Medicine, The First Hospital of Jilin University, Jilin University, Changchun, China; ^2^Chemistry of Traditional Chinese Medicine, College of Pharmacy, Changchun University of Chinese Medicine, Changchun, China; ^3^Institute of Virology and AIDS Research, The First Hospital of Jilin University, Jilin University, Changchun, China; ^4^Department of Experimental Pharmacology and Toxicology, School of Pharmacy, Jilin Univrsity, Changchun, China; ^5^Academy of Integrative Medicine, Dalian Medical University, Dalian, China; ^6^Key Laboratory of Natural Medicines and Immunotechnology of Henan Province, Henan University, Kaifeng, China

**Keywords:** coxsackievirus (CVA6), cell cycle arrest, G0/G1 phase, viral production, non-structural protein

## Abstract

Recent epidemiological data indicate that outbreaks of hand, foot, and mouth disease (HFMD), which can be categorized according to its clinical symptoms as typical or atypical, have markedly increased worldwide. A primary causative agent for typical HFMD outbreaks, enterovirus 71 (EV71), has been shown to manipulate the cell cycle in S phase for own replication; however, it is not clear whether coxsackievirus (CVA6), the main agent for atypical HFMD, also regulates the host cell cycle. In this study, we demonstrate for the first time that CVA6 infection arrests the host cell cycle in G0/G1-phase. Furthermore, synchronization in G0/G1 phase, but not S phase or G2/M phase, promotes viral production. To investigate the mechanism of cell cycle arrest induced by CVA6 infection, we analyzed cell cycle progression after cell cycle synchronization at G0/G1 or G2/M. Our results demonstrate that CVA6 infection promotes G0/G1 phase entry from G2/M phase, and inhibits G0/G1 exit into S phase. In line with its role to arrest cells in G0/G1 phase, the expression of cyclinD1, CDK4, cyclinE1, CDK2, cyclinB1, CDK1, P53, P21, and P16 is regulated by CVA6. Finally, the non-structural proteins of CVA6, RNA-dependent RNA polymerase 3D and protease 3C , are demonstrated to be responsible for the G0/G1-phase arrest. These findings suggest that CVA6 infection arrested cell cycle in G0/G1-phase via non-structural proteins 3D and 3C, which may provide favorable environments for virus production.

## Introduction

Hand, foot, and mouth disease (HFMD) is a febrile exanthematous disease with typical symptoms or atypical symptoms. The typical symptoms of HFMD include flat discolored spots or bumps that may blister, on the hands, feet, and mouth, and occasionally buttocks and groin, even inflammation of the heart, fluid in the lungs, or bleeding into the lungs, inflammation of the brain, inflammation of the meninges, or acute flaccid paralysis, sometimes accompanied by death in children younger than 5 years of age. Epidemiological results show that typical HFMD usually is caused by enterovirus 71 (EV71) or coxsackievirus A16 (CA16) (Chan et al., 2003; Wang et al., 2012). Atypical HFMD, on the other hand, is characterized by severe rash, onychomadesis in young children, and a higher rate of infection in adults, and it is usually induced by coxsackievirus A6 (CVA6). Increasing epidemiological data indicates that outbreaks of CVA6-associated HFMD have markedly increased worldwide in recent years (Gao et al., 2016; Laga et al., 2016; Li J. S. et al., 2016; Li W. et al., 2016; Mirand et al., 2016). However, the current vaccine development, drug development or basic research for HFMD principally targets EV71 and CA16. Therefore, strategies for preventing and treating CVA6-related-HFMD are scarce, and even the current knowledge on the infection, pathogenic mechanism, and immunogenicity of CVA6 remains limited.

As part of their pathogenic mechanism, many viruses are known to control the host cell cycle to maximize their own replication. Examples can be found among DNA viruses, retroviruses, and RNA viruses. Small DNA viruses, such as simian virus 40 (DeCaprio et al., 1988), adenovirus (Howe et al., 1990; Eckner et al., 1994), and human papillomavirus (Werness et al., 1990), as well as large DNA viruses, such as herpesviruses (Flemington, 2001), can arrest the host cell cycle to utilize host resources. The Vpr protein of the retrovirus human immunodeficiency virus type 1 is responsible for accumulating cells in G2/M phase (He et al., 1995; Goh et al., 1998). Among RNA viruses, the coronavirus family virus infectious bronchitis virus (IBV) arrests cells in S and G2/M-phase for viral proliferation (Dove et al., 2006; Li et al., 2007); mouse hepatitis virus (MHV) (Chen and Makino, 2004), severe acute respiratory syndrome coronavirus (SARS-CoV) (Yuan et al., 2005, 2006) and enterovirus 68 (Wang et al., 2017) can induce cell cycle arrest in G0/G1 phase. The etiological agents for typical HFMD, EV71 and CA16, induce cell cycle arrest in S phase for viral production through non-structural protein 3D (Yu et al., 2015). However, whether the atypical HFMD agent, CVA6, regulates the host cell cycle is not clear.

Cell cycle progression in all eukaryotes is controlled by an intricate mechanism that involves CDKs binding with the corresponding cyclin regulatory subunits tightly in sequential order. The G1 phase cyclins, cyclinD, and cyclinE, associate predominantly with CDK4/CDK6 and CDK2 to promote G1 progression and S-phase entry (Sherr, 1994). Up-regulation of P53 and P16 can inhibit the activity of cyclinD/CDK4 or cyclinD/CDK6 complexes to arrest the cell cycle at G0/G1 phase (Sherr and Roberts, 1995). Subsequently, cyclinA and cyclinE combine, mainly with CDK2, to promote S-phase progression (Lundberg and Weinberg, 1998), and then CDK1 and cyclinB regulate mitotic entry (Yu et al., 2008, 2013). Some viruses have been well known to manipulate cell cycle progression by regulating cyclin and CDK expression (Caffarelli et al., 2013; Adeyemi and Pintel, 2014; Yu et al., 2015), but the potential effect of CVA6 on cell cycle regulation is unknown.

In this study, we examined the potential effects of CVA6 infection on the host cell cycle. Our data demonstrate that CVA6 replication arrests cell cycle in G0/G1 phase, and, conversely, that cell cycle arrested in G0/G1 phase provides favorable conditions for CVA6 production. We further demonstrate that the non-structural 3D and 3C and proteins are responsible for G0/G1-phase arrest. These results further advance our understanding of the pathogenic mechanisms of CVA6 and provide a potential target for the treatment and prevention of HFMD.

## Materials and methods

### Cells and viruses

Human embryonic kidney cells (HEK 293T cells) (No CRL-11268) and human rhabdomyosarcoma RD cells (ATCC No CCL-136) were purchased from the ATCC (Manassas, VA, USA). Cells were grown in DMEM (Dulbecco's modified Eagle's medium, Hyclone, Logan, UT, USA) supplemented with 10% FBS (fetal bovine serum, GIBCO BRL, Grand Island, NY, USA). The 46 strain of CVA6 (https://www.ncbi.nlm.nih.gov/nuccore/1071451803) was provided by the Jilin Provincial Center for Diseases Control and Prevention (Changchun, China). Viruses were propagated in RD cells, and the supernatants were collected and stored at –80°C as our previous study (Yu et al., 2015; Wang et al., 2017).

### Plasmid construction and transfection

As our previous studies (Yu et al., 2015; Wang et al., 2017), Trizol (Invitrogen) was used to extract total RNA. According to the supplier's instructions, High-capacity cDNA Reverse Transcription Kit (Applied Biosystems) was used to generate the cDNA with the oligo-d(T)18 primers (Takara). With the primers shown in Table 1, the 3D and 3C gene were cloned using the product from the reverse transcription reaction as the template. The forward primer with HA tag and both primers with restriction sites were engineered. The RT-PCR products were subsequently digested by corresponding restriction enzymes and cloned into corresponding restriction sites of plasmid VR1012 or PEGFP-C1.

**Table 1:** The primer for real time PCR and plasmid construct.

**Primer pair**	**Role**	**Forward sequence5′-3′(restriction enzyme)**	**Reverse sequence5′-3′(restriction enzyme)**
3D	Plasmid construction	AACTGCAGACCATGTACCCTTACGACGTCCCAGATT ACGCGGGAGAGATCCAATGGGTCAAAC (PstI)	CGGGATCCCTAAAATAATT CGAGCCAATTGCG(BamHI)
3C	Plasmid construction	AACTGCAGACCATGTACCCTTACGACGTCCCAGATT ACGCGGGCCCAAGTCTCGACTTT(PstI)	CGGGATCCCTATTGCTCAC TAGCAAAATAGC(BamHI)
VP 1	Real time	AATGAGGCGAGTGTGGAAC	AGGTTGGACACAAAAGTGAACT
GAPDH	Real time	GCAAATTCCATGGCACCGT	TCGCCCCACTTGATTTTGG

### Transfection

According to our previous studies (Yu et al., 2015; Wang et al., 2017), 4 μg of plasmid (including empty vector, 3C and 3D) with 12 μL of Lipofectamine 2000 (Invitrogen) were transfected into 293T cells in a 6-cm cell cultural dishes, for the dose-dependence experiments with VR1012-3D and VR1012-3C, we replenished the empty plasmid VR1012 to maintain a final concentration of 4 μg plasmid/well. At 36 h after transfection, the cells were collected for cell cycle analysis.

### Viral titer determination

As described (Zhong et al., 2017), the viral titer in a microtitration assay was determined by measuring the TCID_50_ in RD cells. Virus was serially prepared after 10-fold dilution, and 100 μL virus/well was inoculated in octuplicate in 96-well plates. The cytopathic effect was measured once per day until the experimental endpoint was reached. The TCID_50_ was determined according to the Reed-Muench method (Reed and Muench, 1983) based on that viruses of 1 × 10^5^ TCID_50_/mL will produce 0.7 × 10^5^ plaque forming units/mL as our previous studies (Reed and Muench, 1983; Yu et al., 2015).

### Infection

Cells were mock-infected or infected with CVA6 at an MOI of 1. After 2 h of virus absorption, cells were washed once with PBS, and cells were cultured with fresh culture medium.

### Cell cycle release

For synchronizing cells in G0/G1 phase, RD cells were deprived serum for 48 h (He et al., 2010). Approximately 5 × 10^5^ cells/well in a 6-well plate were maintained in serum-free medium for 48 h. After virus infection, fresh 10% DMEM was added to release the cells from G0/G1 as our previous studies (Yu et al., 2015; Wang et al., 2017).

### Synchronization of cells

For G0/G1 arrest, RD cells were synchronized by serum deprivation for 24 h (He et al., 2010). For S-phase synchronization, cells were treated with 0.85 mM thymidine (Sigma) (Helt and Harris, 2005) for 24 h. For G2/M arrest, RD cells were treated with 25 ng/mL nocodazole (Sigma) (Wang et al., 2017) for 24 h. For sustained corresponding cell-cycle arrest after virus infection, fresh medium of serum-free, fresh medium of 0.85 mM thymidine or fresh medium of 25 ng/mL nocodazole were re-treated for the indicated times as our previous studies (Yu et al., 2015; Wang et al., 2017).

### Cell cycle analysis by flow cytometry

According to our previous studies (Yu et al., 2015; Wang et al., 2017), cellular DNA content was measured by fluorescence-activated cell sorting (FACS) after propidium iodide (PI) staining.

### Western blot analysis

As our previous studies (Yu et al., 2015; Wang et al., 2017), virus-infected and mock-infected cells were collected at indicated times. The following antibodies were used: anti-cyclinE1 (Proteintech), anti-CDK2 (Cell Signal), anti-cyclinD (Cell Signal), anti-CDK6 (Cell Signal), anti-CDK4 (Cell Signal), anti-P53 (Cell Signal), anti-P21 (Proteintech), anti-P16 (Proteintech), anti-cyclinB1 (Santa Cruz), anti-CDK1 (Boster), anti-VP1 (Genetex) and anti-histone (GenScript). Secondary antibodies from mouse or rabbit were obtained from Jackson Immuno Research.

### Quantitative real-time RT-PCR

According to our previous studies (Yu et al., 2015; Wang et al., 2017), intracellular viral genome RNA was detected through targeting VP1 primers (Table 1). The fold changes were calculated relative to GAPDH using the ΔΔCt method for VP1.

### Statistical analyses

Statistical differences were analyzed using the Student's *t*-test for all analyses, except for the 3C and 3D dose-dependent test, for which the Pearson correlation coefficient was used. Data are presented as means and standard deviations (SD). ^*^P-values of < 0.05 were considered statistically significant.

## Results

### Cells accumulate in G0/G1 phase after CVA6 infection

**Figure 1:**
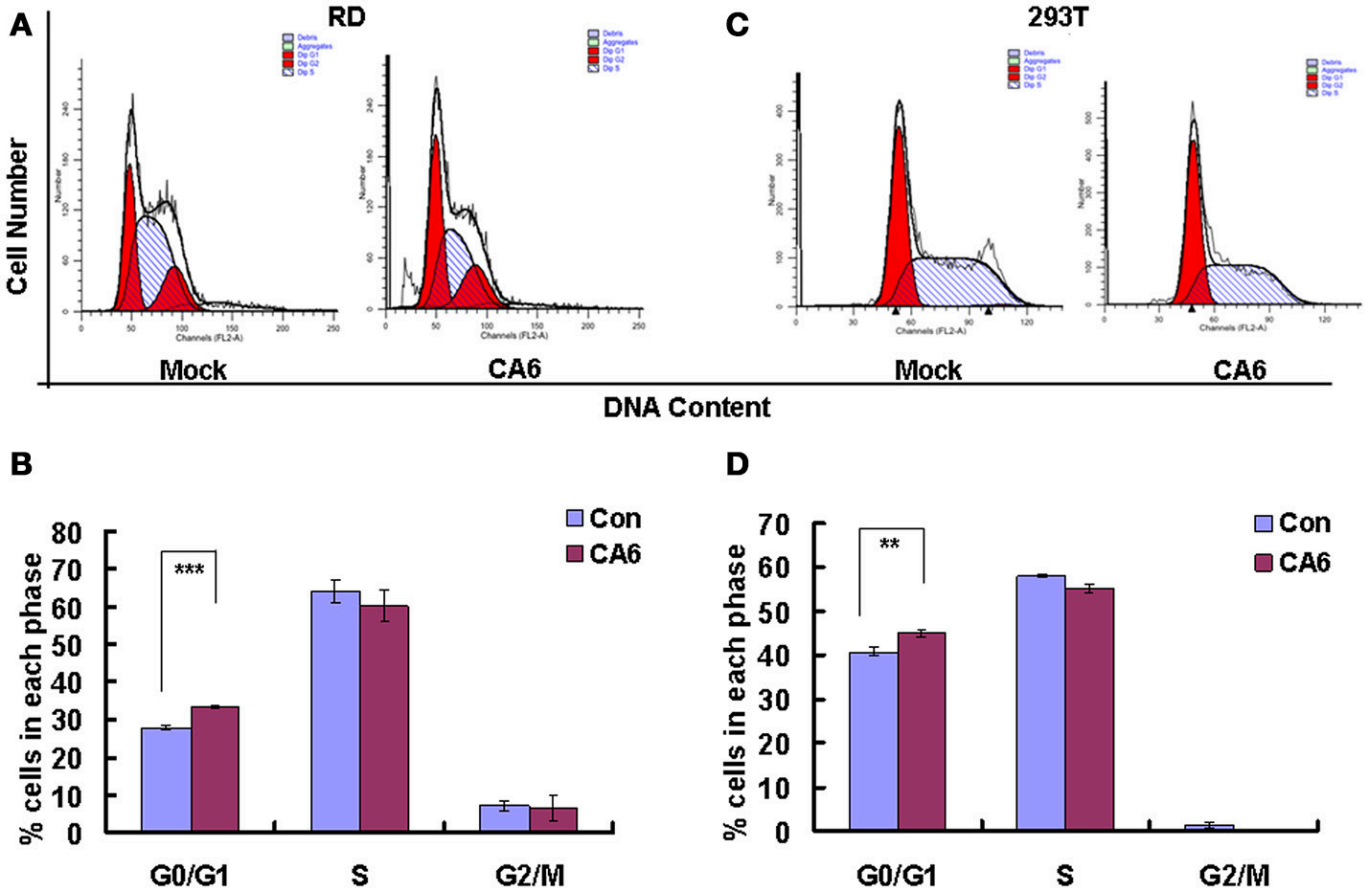
CVA6 infection induces G0/G1-phase accumulation**. (A)** At 24 h post-infection, RD cells infected with mock (Mock) or with CVA6 (CVA6) at an MOI of 1 were collected for analyzing cell-cycle profiles by flow cytometry. **(B)** The histograms were analyzed by the ModFit LT program to display the cell cycle distribution. ^***^P < 0.001. **(C)** At 48 h post-infection, 293T cells infected with mock (Mock) or with CVA6 (CVA6) at an MOI of 5 were collected for analyzing cell-cycle profiles by flow cytometry. **(D)** The histograms indicating cell cycle distribution were analyzed by the ModFit LT program. ^**^P < 0.01. The results indicate the mean ± SD of three independent experiments.

To assess whether CVA6 manipulated the cell cycle of host cells, the cell cycle distribution by flow cytometry was analyzed in human rhabdomyosarcoma RD cells at 24 h post-infection. An obvious increase in the G0/G1 phase was observed by ModFit analysis, with the increase from 27.66 ± 0.52% for mock-infected cells to 33.37 ± 0.40% for CVA6-infected (20.64% increase; *P* < 0.001;). These data suggest that CVA6 infection induces G0/G1-phase accumulation. Meanwhile, to determine whether or not G0/G1-phase arrest is exclusive to the RD cell line, human embryonic kidney cells 293T were selected for further analysis based on screening cell line with cytopathic effect after CVA6 infection. 293T cells in G0/G1 phase were increased from 40.80 ± 1.05 to 44.89 ± 0.95% (10.02% increase; *P* < 0.00–1; Figure 1B) at 48 h post-infection, and it was found that cytophathic effect induced by CVA6 in 293T is not obvious as RD cell line (data not shown), which might explain that CVA6 manipulated cell cycle in 293T cell line not as strongly as in RD cell line. These results indicate that the effects of CVA6 on G0/G1-phase arrest are broadly applicable.

### G0/G1-phase arrest promotes the production of CVA6

**Figure 2:**
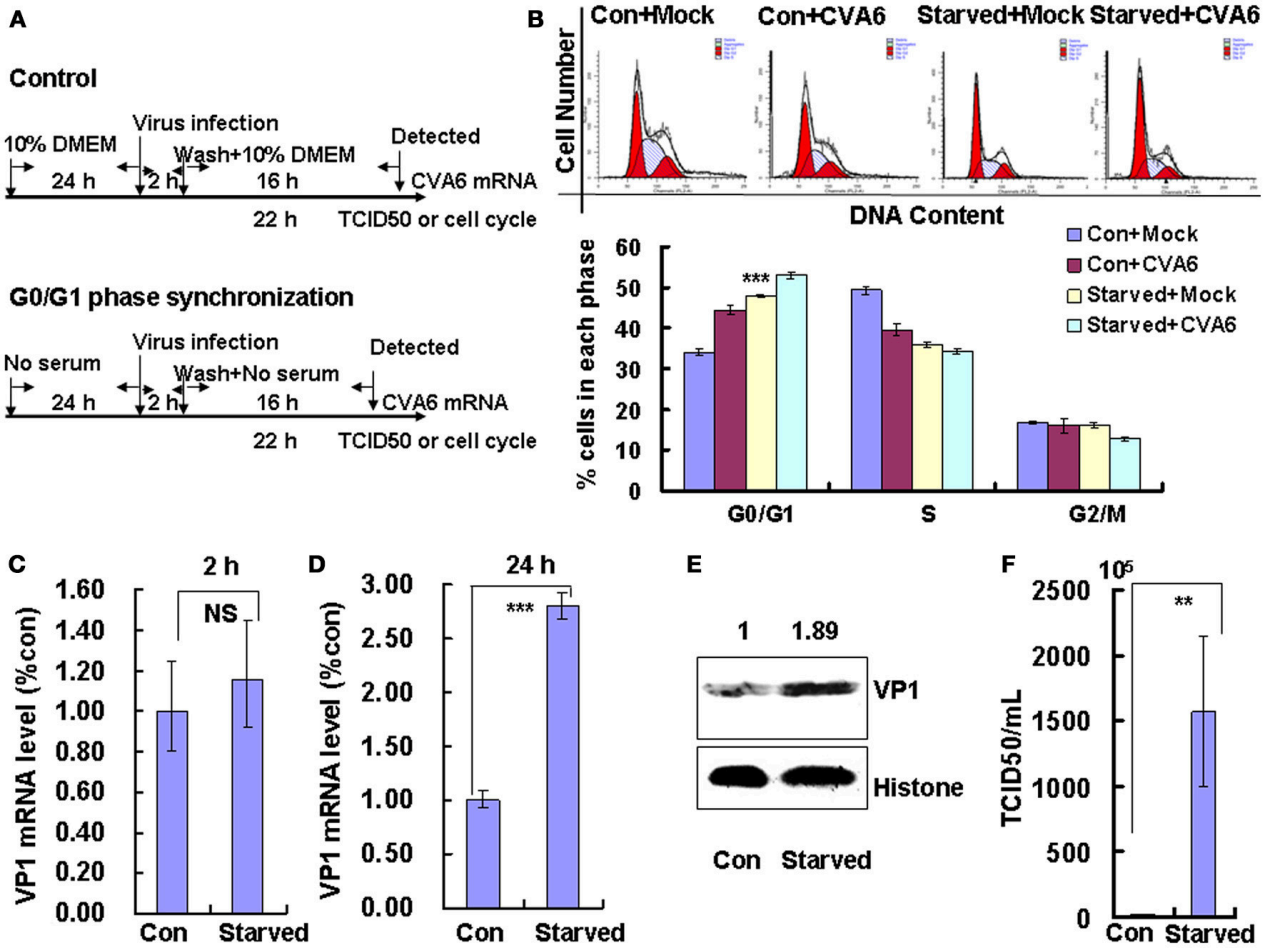
G0/G1 phase-synchronization promotes viral replication. **(A)** RD cells were cultured in serum-free medium for 24 h for G0/G1-phase synchronization. Infected with mock (Mock) or infected with CVA6 (CVA6) at an MOI of 1 for 2 h, then the medium was restored to maintain the cell cycle synchronization status for 24 h. **(B)** Top panel: Flow cytometry determined the cell cycle profiles after culture in control medium (Con) or serum-free medium (Starved) and mock-infection or infection with CVA6. Bottom panel: The histograms indicating cell cycle distribution were analyzed by the ModFit LT program. ^***^P < 0.001 (Starved+Mock vs. Con+Mock). **(C,D)** Intracellular CVA6 RNA levels were detected by quantitative real-time PCR in RD cells that were cultured in control (Con) or serum-free medium (Starved) at 2 h **(C)** or 18 h **(D)** post-infection with CVA6. The results were standardized using GAPDH mRNA as a control and normalized to 1.0 in the Con cells. **(E)** The expression of VP1 was determined in control medium (Con) or serum-free medium (Starved)-treated cells at 24 h post-infection by Western blot analysis. Histone is the loading control. The results are representative of three independent experiments. **(F)** The total progeny viruses (the supernatant and intracellular viruses) were titrated in RD cells, and the TCID_50_/mL value was determined at 24 h post-infection. The results represent the mean ± SD of three independent experiments. NS, no significant difference; ^**^P < 0.01 and ^***^P < 0.001.

The above data indicate that CVA6 infection induces cell cycle arrest in G0/G1 phase; however, it is still unknown whether this viral strategy is actually beneficial to the virus. To explore the possible benefits of G0/G1-phase arrest for viral replication, the cells were synchronized in G0/G1 phase by culture in serum-free medium (Figure 2A). In the absence of infection, 48 h serum starvation increased the ratio of G0/G1 cells from 33.48 ± 0.74 to 47.95 ± 0.25% (*P* < 0.001, Starved+Mock vs. Con+Mock), which verifies that the cells were properly synchronized in G0/G1 phase (Figure 2B). Furthermore, in the absence of serum starvation, CVA6 infection induced G0/G1 arrest at 24 h post infection from 33.48 ± 0.74 to 44.43 ± 1.14% (*P* < 0.001, Con+CVA6 vs. Con+Mock), which is consistent with the results for Figure 1. Additionally, in the absence of serum, CVA6 infection for 24 h further increased the ratio of G0/G1 cells to 52.94 ± 0.68% (*P* < 0.001, Starved+CVA6 vs. Con+CVA6), indicating that CVA6 infection increases the G0/G1 phase arrest caused by serum starvation.

To directly assess the effects of serum starvation on the viral RNA levels, we performed quantitative real-time PCR (qRT-PCR) of the VP1 gene in the absence and presence of serum starvation. At 2 h post-infection (viral entry stage), the CVA6 genomic levels were not obviously different in the control and serum-starved cells (*P* > 0.05, Starved vs. Con;); however, at 18 h post infection (viral replication stage) 2.802 times more viral genomic RNA was discovered in the serum-starved cells than in the control cells (*P* < 0.001, Starved vs. Con; Figure 2D). Furthermore, at 24 h post infection (viral production stage), the VP1 viral protein levels were increased 1.89-fold in the serum-starved cells as compared to the control cells (*P* < 0.001, Starved vs. Con; Figure 2E). Additionally, at 24 h the TCID_50_/mL of infectious CVA6 particles was 57.72 times higher for total viruses including supernatant and intracellular viruses from G0/G1 phase-synchronized cells (1.57 ± 0.58 × 10^8^) than from control cells (2.72 ± 0.39 × 10^6^) (*P* < 0.01, Starved vs. Con; Figure 2F). The results indicate that G0/G1-phase synchronization does not affect viral entry, but promotes CVA6 production.

### S- and G2/M-phase synchronization do not facilitate viral production

**Figure 3:**
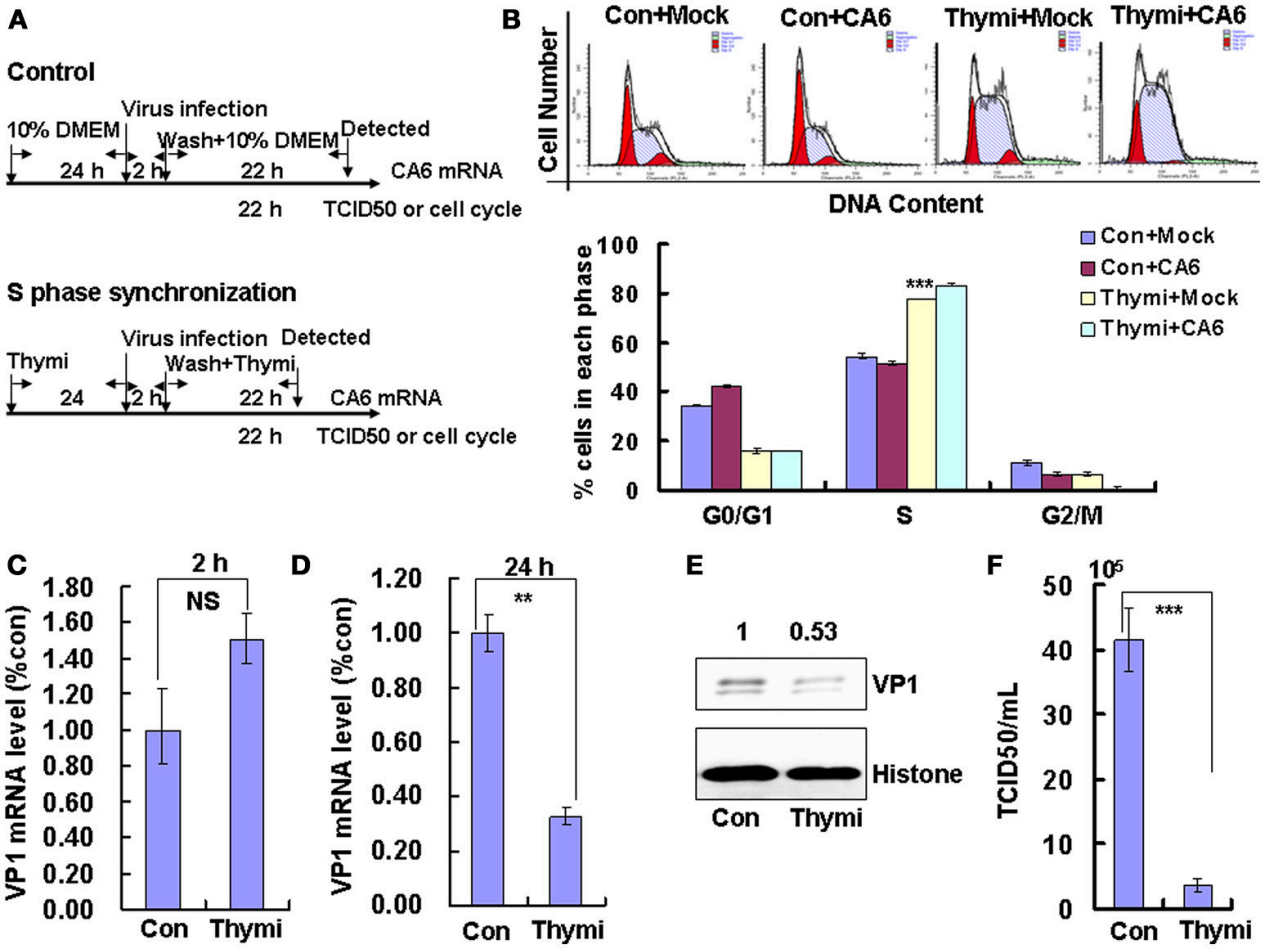
S phase-synchronization inhibits viral production. **(A)** RD cells were treated without (Con) or with 0.85 mM thymidine (Thymi) for 24 h for S phase synchronization. Then, RD cells were infected with mock (Mock) or with 1 MOI of CVA6 (CVA6), after 2 h infection, and cells was re-treated with fresh culture medium or 0.85 mM thymidine for another 24 h. **(B)** Top panel: Flow cytometry determined the cell cycle profiles after culture in control medium (Con) or thymidine (Thymi)-containing medium for 24 h. Bottom panel: The histograms indicating the cell cycle distribution were analyzed by the ModFit LT program. ^***^P < 0.001 (Thymi+Mock vs. Con+Mock). **(C,D)** Intracellular CVA6 genomic RNA levels were detected in RD cells with or without thymidine by quantitative real-time PCR at 2 h **(C)** or 24 h **(D)** post-infection. The results were standardized using GAPDH mRNA as a control and normalized to 1.0 in Con cells. **(E)** The expression of VP1 was determined in cells cultured in control medium (Con) or thymidine medium (Thymi) at 24 h post-infection by Western blot analysis. Histone is the loading control. The results are representative of three independent experiments. **(F)** The total progeny viruses (the supernatant and intracellular viruses) were titrated in RD cells, and at 24 h post-infection the TCID_50_/mL value was determined. The results represent the mean ± SD of three independent experiments. NS: no significant difference, ^**^P < 0.01 and ^***^P < 0.001.

To determine whether the cell-cycle-arrest-dependent enhancement of CVA6 viral replication is specific for G0/G1-phase arrest, the effect of S and G2/M arrest on viral replication is assessed. To induce S phase synchronization, thymidine were treated in cultured cells (Figure 3A). It was found that 0.85 mM thymidine induced obvious S phase arrest compared to control treatment (*P* < 0.001). However, CVA6 was unable to accumulate cells in G0/G1 phase that had been treated with thymidine (Figure 3B). The viral genomic RNA levels, as assessed by VP1 qRT-PCR remained similar in S phase-synchronized cells and control non-synchronized cells at 2 h post-infection (Figure 3C) but were reduced in S phase-synchronized cells at 24 h post-infection (*P* < 0.01, Thymi vs. Con; Figure 3D). Consistently, the expression of VP1 protein in S phase-synchronized cells was lower than that in control non-synchronized cells at 24 h post-infection (*P* < 0.01, Thymi vs. Con; Figure 3E). Furthermore, the values of TCID_50_/mL at 24 h post-infection were much lower for total viruses including supernatant and intracellular viruses in the S phase-synchronized cells (3.67 ± 0.89 × 10^5^) than in the control cells (41.4 ± 4.86 × 10^5^) (*P* < 0.001; Figure 3F). These results indicate that arrest in S-phase does not affect CVA6 entry, but inhibits viral production.

**Figure 4:**
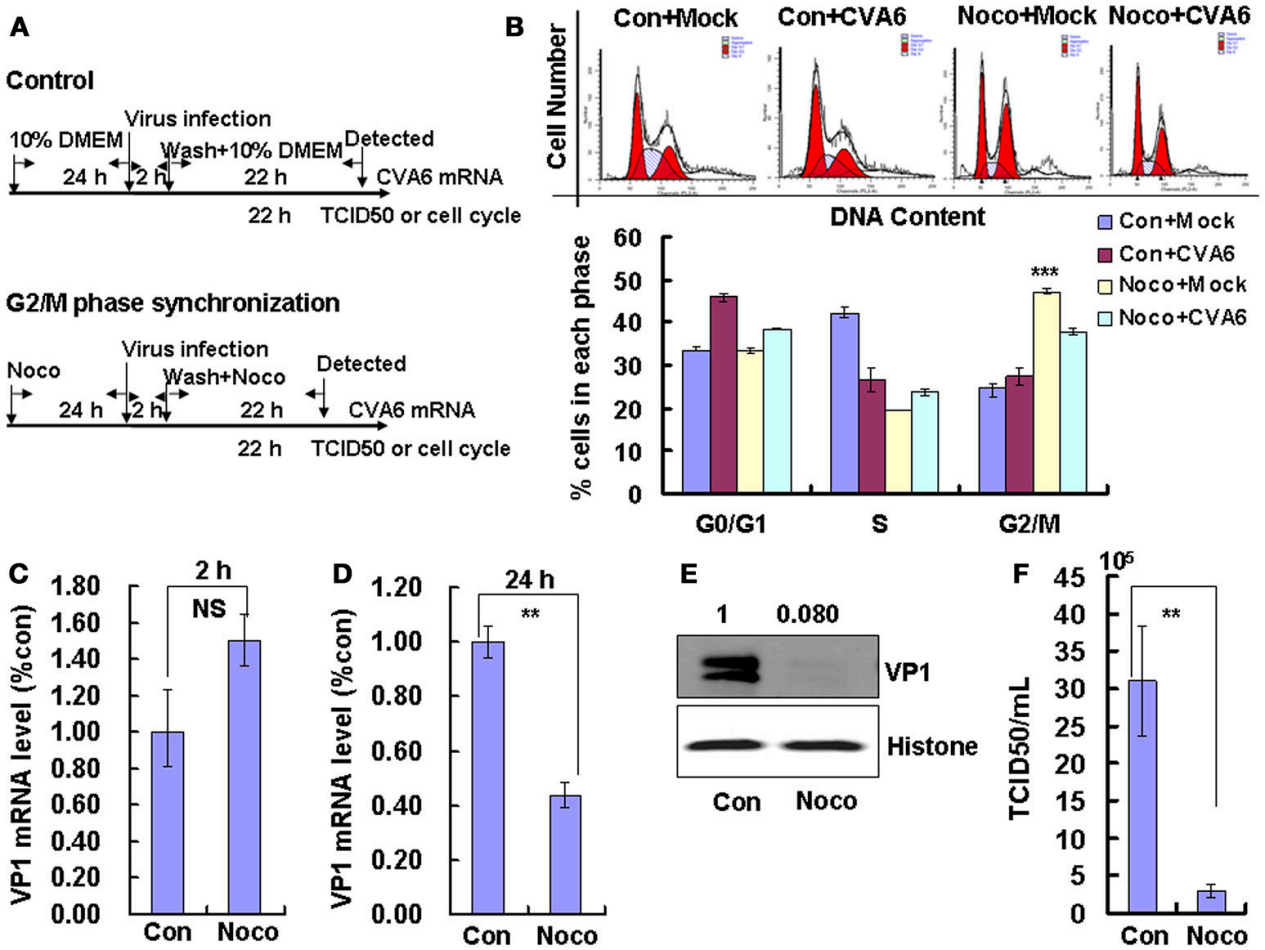
G2/M-phase synchronization by nocodazole inhibits viral production. **(A)** For G2/M synchronization, RD cells were cultured in control medium (Con) or 25 ng/mL nocodazole (Noco) for 24 h. Then cells were infected with mock (Mock) or CVA6 (CVA6) at an MOI of 1 for 2 h, and then fresh control medium or fresh 25 ng/mL nocodazole were treated for another 24 h. **(B)** Top panel: Flow cytometry determined the cell cycle profiles after culture in control medium (Con) or nocodazole (Noco)-containing medium for 24 h. Bottom panel: The histograms indicating the cell cycle distribution were analyzed by the ModFit LT program. ^***^P < 0.001 (Noco+Mock vs. Con+Mock). **(C,D)** By quantitative real-time PCR intracellular CVA6 RNA levels were detected in RD cells with or without nocodazole treatment at 2 h **(C)** and 24 h **(D)** post-infection. The results were standardized using GAPDH mRNA as a control and normalized to 1.0 in Con cells. **(E)** By Western blot analysis, the expression of VP1 was determined after culture in control medium (Con) or nocodazole medium (Noco) at 24 h post-infection. Histone is the loading control. The results are representative of three independent experiments. **(F)** At 24 h post-infection, TCID_50_/mL of the total progeny viruses (the supernatant and intracellular viruses) was detected in RD cells. The results represent the mean ± SD of three independent experiments. NS, no significant difference; ^**^P < 0.01.

To induce G2/M phase synchronization, cells were treated with nocodazole (Figure 4A). Nododazole obviously accumulated cells in G2/M phase (*P* < 0.001; Figure 4B). Furthermore, at 48 h after nocodazole treatment, the G2/M percentage for mock-infected cells at (47.20 ± 0.70%) was greater than the G2/M percentage for CVA6-infected cells (37.81 ± 0.85%); however, the G0/G1 percentage for mock-infected cells after nocodazole treatment for 48 h (33.28 ± 0.62%) was less than the G0/G1 percentage for CVA6-infected cells after nocodazole treatment (38.46 ± 0.15%; *P* < 0.001; Figure 4B). These results suggest that CVA6 infection promotes G0/G1-phase entry from the G2/M phase.

We also examined the effects of nocodazole on genomic RNA levels. At 2 h post-infection, the genomic RNA level in G2/M phase-synchronized cells was not different from the control non-synchronized cells (Figure 4C); however, at 24 h post-infection the genomic level in the synchronized cells was significantly lower than in the control cells (*P* < 0.01; Figure 4D). The VP1 expression in G2/M phase-synchronized cells was lower than in control cells at 24 h post-infection (*P* < 0.01; Figure 4E). Meanwhile, the TCID_50_/mL of total viruses including supernatant and intracellular viruses was obviously lower in the G2/M phase-synchronized cells (2.95 ± 0.91 × 10^5^) than that in the control non-synchronized cells (3.10 ± 0.74 × 10^6^) at 24 h post-infection (*P* < 0.01; Figure 4F). Therefore, these results indicate that synchronization in G2/M phase does not affect viral entry, but decreases CVA6 production. Therefore, the enhancement of CVA6 replication upon cell cycle arrest is specific for the G0/G1 phase.

**Figure 5:**
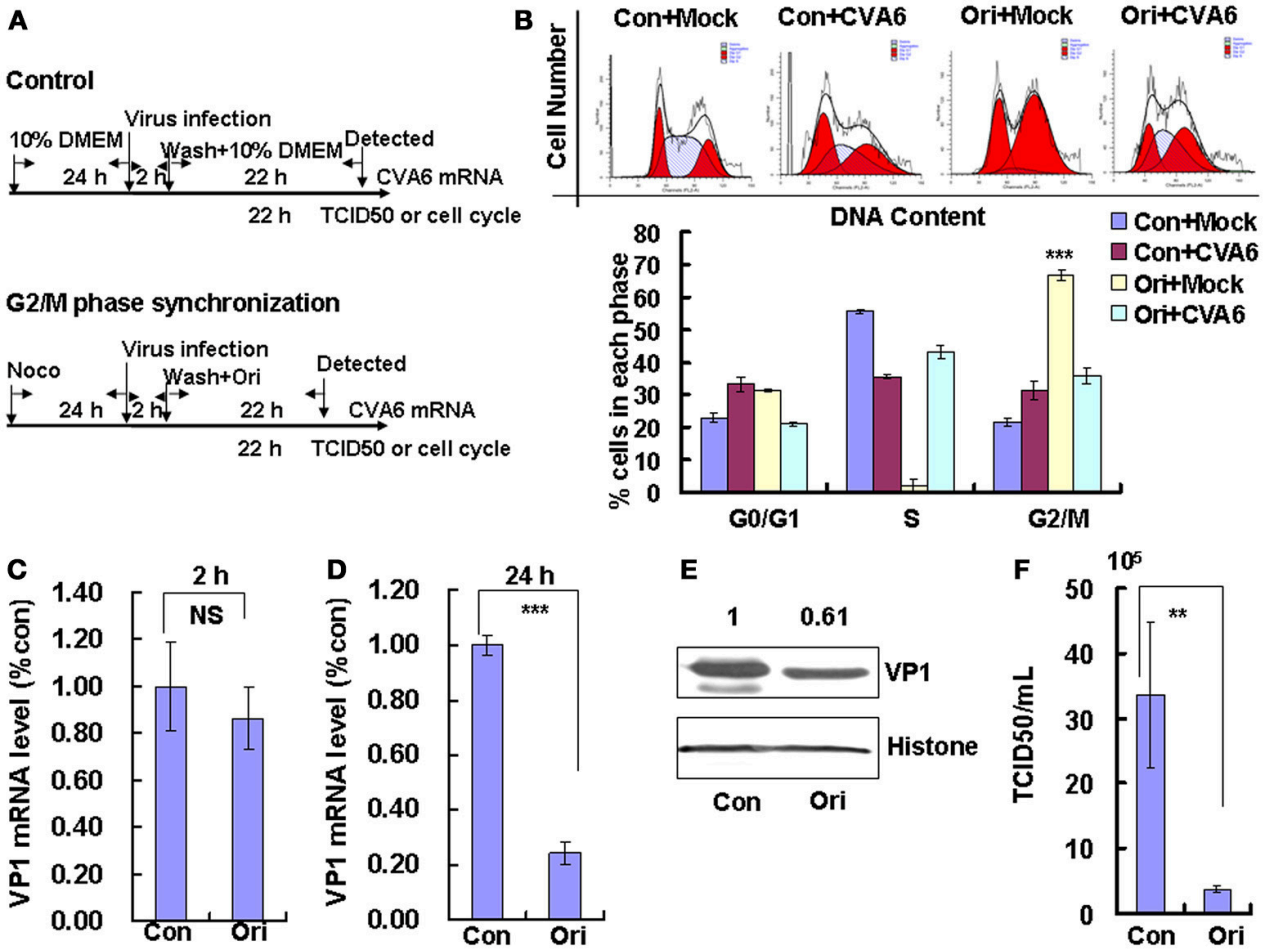
G2/M-phase synchronization by Oridonin inhibits viral production. **(A)** RD cells were treated with or without 8 μM Oridonin (Ori) for 24 h, infected with mock (Mock) or CVA6 at an MOI of 1 for 2 h, and then treated again with or without 8 μM Oridonin for 24 h. **(B)** Top panel: Flow cytometry determined the cell cycle profiles after growth in control medium (Con) or Oridonin (Ori)-containing medium for 24 h. Bottom panel: The histograms indicating the cell cycle distribution were analyzed by the ModFit LT program. **(C,D)** By quantitative real-time PCR, CVA6 RNA levels were detected at 2 h **(C)** and 24 h **(D)** post-infection in G2/M phase-synchronized or non-synchronized cells. The results were standardized using GAPDH mRNA as a control and normalized to 1.0 in Con cells. ^***^P < 0.001. **(E)** By Western blot analysis, the expression of VP1 was determined after culture in control medium (Con) or Oridonin medium (Ori) at 24 h post-infection. Histone is the loading control. The results are representative of three independent experiments. **(F)** At 24 h post-infection, the TCID_50_/mL value of total progeny viruses (the supernatant and intracellular viruses) was determined in RD cells. The results represent the mean ± SD of three independent experiments. NS, no significant difference; ^**^P < 0.01.

Followed that, Oridonin, which is an active diterpeniod isolated from Rabdosia rubescens, was utilized to confirm the effects of G2/M-related drugs on viral production. Oridonin induces cell cycle arrest in the G2/M phase through inducing DNA damage (Zhang et al., 2013; Zheng et al., 2017), which is different from nocodazole through interfering with the polymerization of microtubules. To induce G2/M phase synchronization, cells were treated with 8 μM Oridonin (Figure 5A). Oridonin obviously accumulated cells in G2/M phase (*P* < 0.001; Figure 5B). We also examined the effects of Oridonin on genomic RNA levels. At 2 h post-infection, the genomic RNA level in the control cells was not obvious different with that in G2/M phase-synchronized cells (Figure 5C); however, at 24 h post-infection the genomic level in the G2/M synchronized cells was lower than in the control non-synchronized cells (*P* < 0.001; Figure 5D). The expression of VP1 protein in Oridonin-treated cells was lower than in control cells at 24 h post-infection (*P* < 0.05; Figure 5E). Meanwhile, the TCID_50_/mL for total viruses including supernatant and intracellular viruses from the G2/M phase-synchronized cells (0.38 ± 0.52 × 10^5^) was obviously lower than that from the control non-synchronized cells (3.36 ± 1.11 × 10^6^) at 24 h post-infection (*P* < 0.01; Figure 5F). Therefore, it is concluded that G2/M synchronization does not affect viral entry, but decreases CVA6 production. Therefore, the enhancement of CVA6 replication upon cell cycle arrest is specific for the G0/G1 phase.

### CVA6 infection prevents entry into the S phase

**Figure 6:**
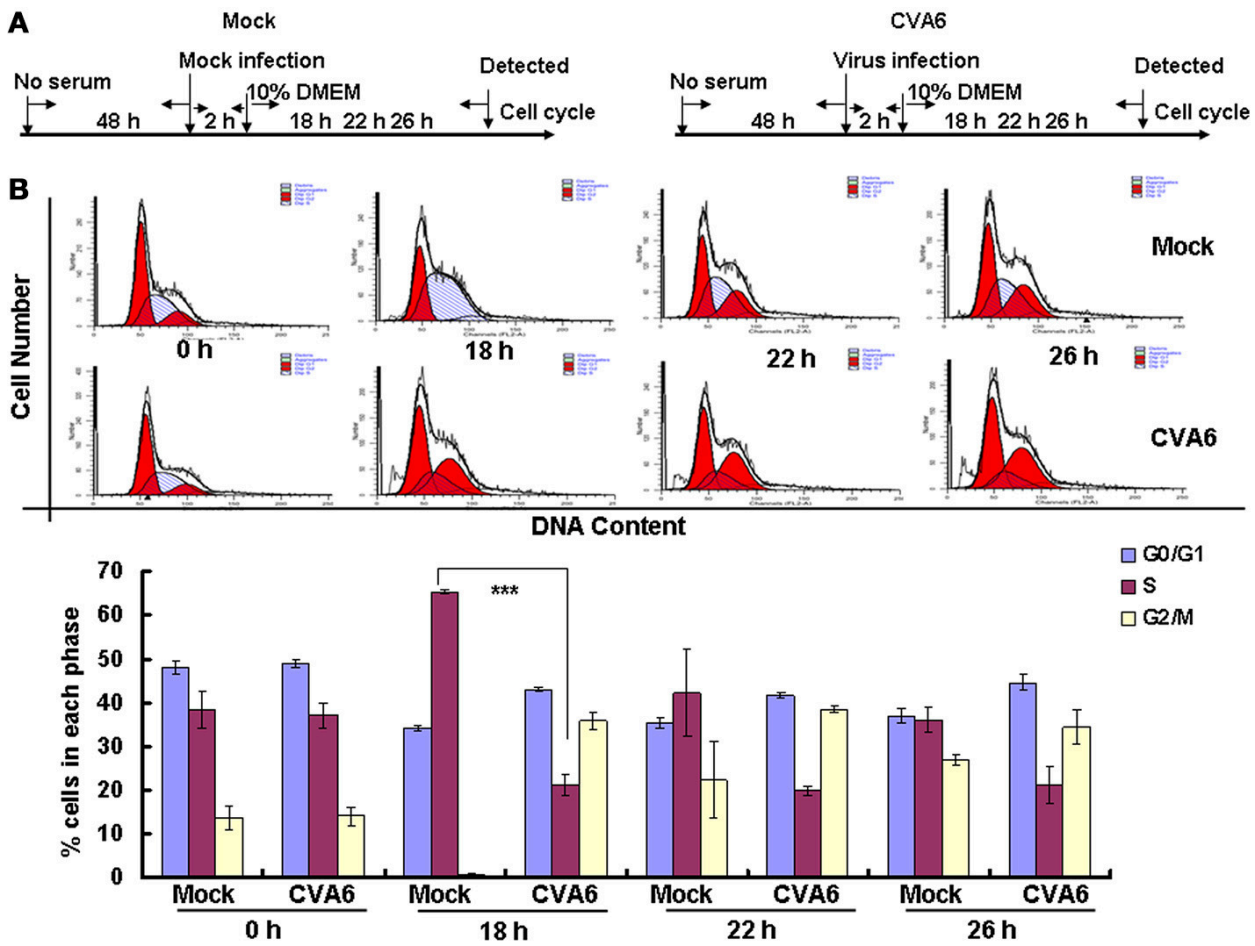
CVA6 infection prevents cell exit from G0/G1 into S phase. **(A)** RD cells were cultured in serum-free medium for 48 h and then mock-infected (Mock) or infected with CVA6 (CVA6) at an MOI of 1. After 2 h of virus absorption, the cells were treated with medium containing 10% FBS. **(B)** Top panel: Flow cytometry determined the cell cycle profiles at 0, 18, 22, and 26 h post-infection. Bottom panel: The histograms indicating the cell cycle distribution were analyzed by the ModFit LT program. The results represent the mean ± SD of three independent experiments. ^***^P < 0.001.

The above results suggest that CVA6 may induce G0/G1 arrest in part by promoting G2/M exit. To determine whether CVA6 also may achieve G0/G1 arrest by preventing G0/G1 phase entry into S phase, by serum starvation RD cells were synchronized in G0/G1 phase, then with 10% FBS to trigger cell cycle re-entry (Figure 6A). After 18 h of serum stimulation, the mock-infected cells entered into S phase and then gradually owned the cell cycle distribution as normal cells. However, CVA6-infected RD cells remained in G0/G1 phase after 18 h of serum stimulation and remained in G0/G1 phase throughout the 26 h time course (Figure 6B). Therefore, CVA6 infection prevents cells entry into S phase from G0/G1 phase.

### Key cell cycle regulatory molecules are regulated by CVA6 infection

**Figure 7:**
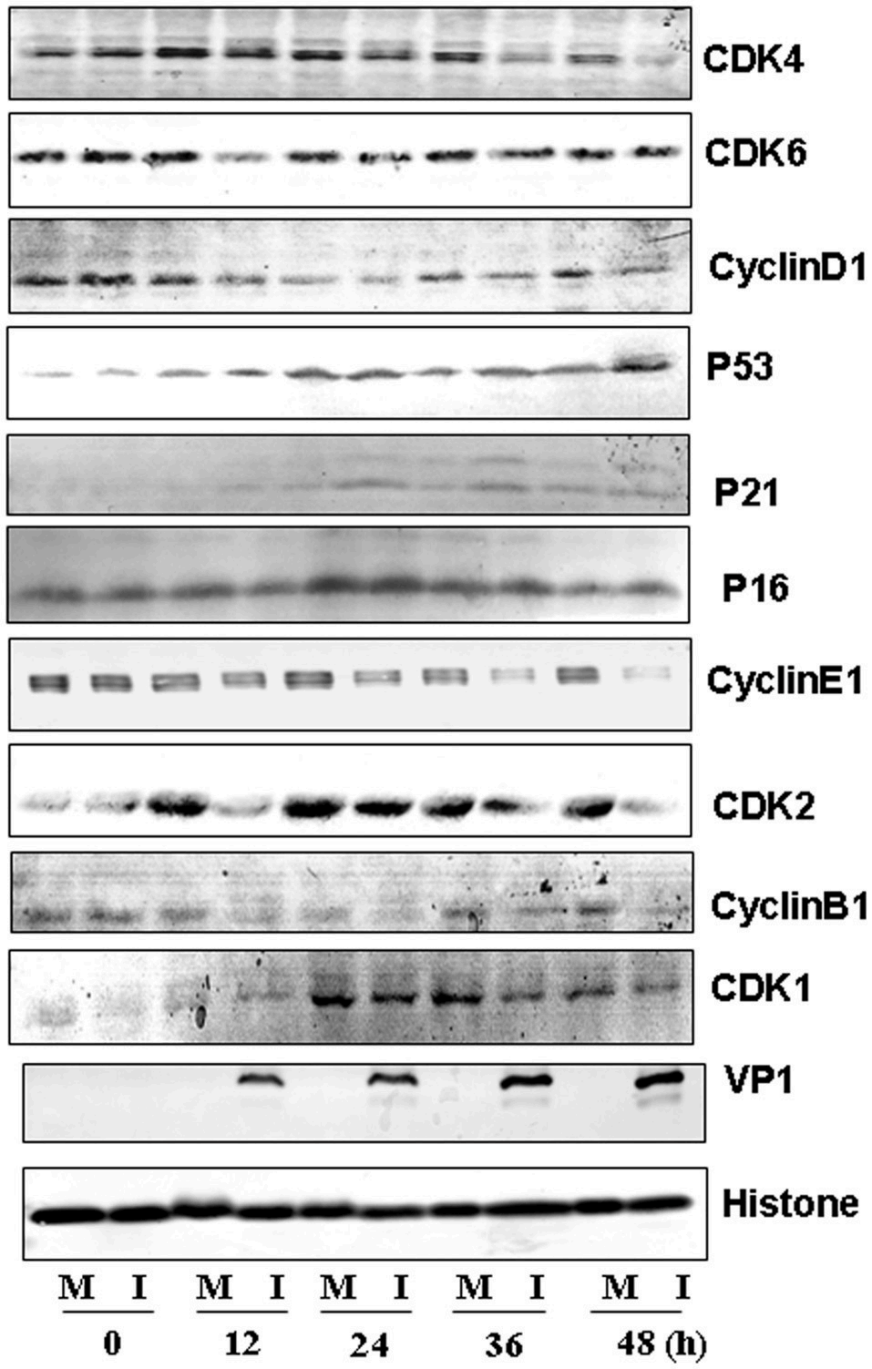
Western blot analysis of cell-cycle related proteins. RD cells, mock-infected (M) or infected with CVA6 (I) at an MOI of 1, were collected at 0, 12, 24, 36, and 48 h. The expression of CDK4, CDK6, cyclinD1, P53, P21, P16, cyclinE1, CDK2, cyclinB1, CDK1, and VP1 proteins was analyzed. Histone is the loading control. The results were representative of three independent experiments.

To further understand the molecular signaling pathways that are modulated by CVA6 infection, we collected RD cells at 0, 12, 24, 36 and 48 h post-infection for the expression of G0/G1-phase related proteins by Western blotting. Consistent with ability of CVA6 to mediate G0/G1 arrest, the expression of CDK4 and CDK6, which have been established to mediate G0/G1 cell cycle transition to S phase (Sherr, 1994), was decreased at 12 h post-infection, and the expression of the CDK4/CDK6-interacting partner, cyclinD1 was decreased at 36 h post-infection (Figure 7, first three rows). Furthermore, the expression of P53, P21 and P16, which are known to inhibit CDK4/6 function (Massagué, 2004), was increased in CVA6-infected cells comparing with mock-infected cells at 24 h post-infection (Figure 7, next three rows). These results indicate that both the P53-P21 pathway and the P16 pathway are activated by CVA6 infection to regulate CDK4/cyclinD1 or CDK6/cyclinD1 and arrest the cells in G0/G1 phase.

We also evaluated the expression of cyclinE1 and CDK2, which promote cell cycle progression from G0/G1 phase to S phase (Darzynkiewicz et al., 1980) and cyclinB1 and CDK1, which are involved into mitotic progression (Coverley et al., 2002; Yam et al., 2002). The expression of CDK2 and cyclinE1 was decreased after 12 h post-infection, and the expression of cyclinB1 and CDK1 was decreased by CVA6 infection at all time point tested (Figure 7, last six rows). These findings are consistent with the ability of CVA6 to promote G2/M exit into G0/G1 phase.

### The viral non-structural proteins 3D and 3C of CVA6 are responsible for G0/G1-phase arrest

**Figure 8:**
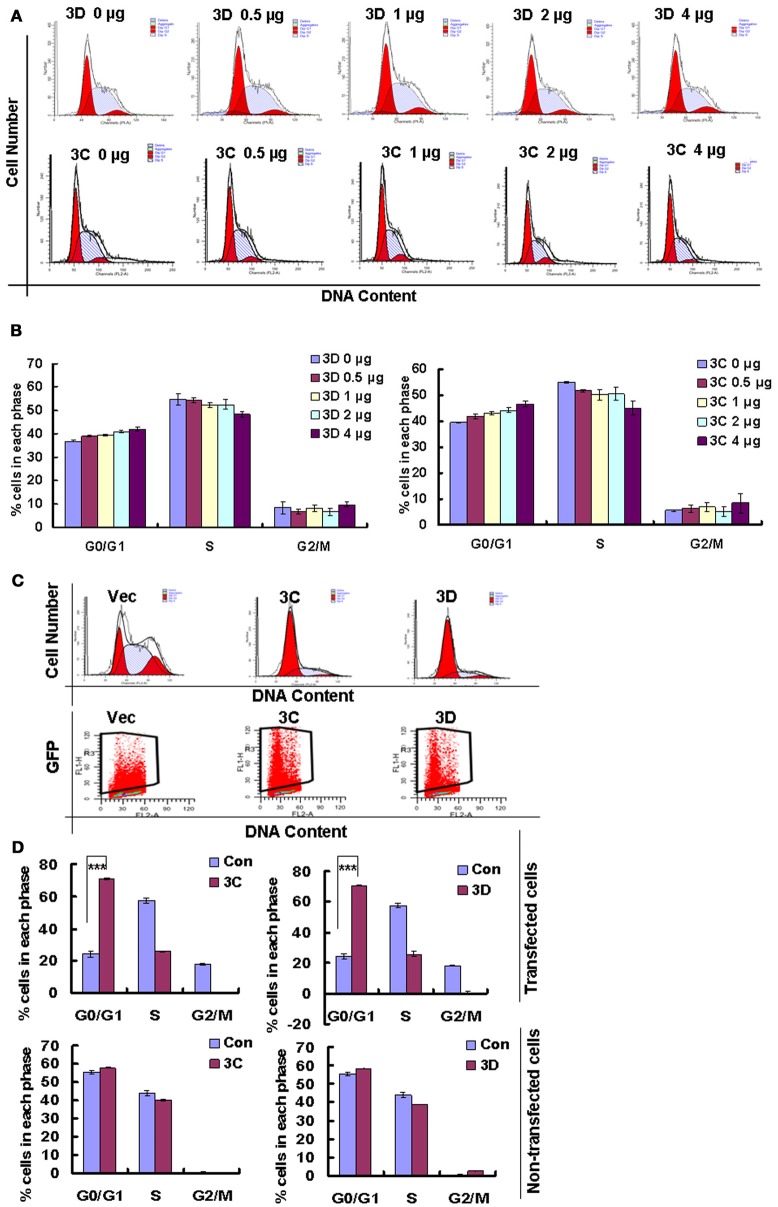
The effect of non-structural proteins 3D and 3C on G0/G1-phase arrest. **(A)** The cell cycle distribution in 293T cells was analyzed at 36 h after transfection with 0, 0.5, 1, 2, or 4 μg plasmid as indicated. **(B)** The histograms indicating the cell cycle distribution were analyzed by the ModFit LT program. The results indicate the mean ± SD of three independent experiments. **(C)** The cell cycle distribution and fluorescence density in 293T cells were analyzed at 36 h after transfection with 4 μg PEGFP (Vec), 3C-PEGFP (3C) and 3D-PEGFP (3D) plasmid. **(D)** The histograms displaying the cell cycle distribution were analyzed by the ModFit LT program. Top panel: Cells with green fluorescence indicated successful transfection. Bottom panel: Cells without green fluorescence indicated non-successful transfection. The results indicate the mean ± SD of three independent experiments. ^***^P < 0.001.

On the base of the established role of viral non-structural proteins in cell-cycle manipulation from other viruses (Goh et al., 1998; Yuan et al., 2006), we assessed whether the exogenous expression of the non-structural proteins 3D and 3C of CVA6 might mediate G0/G1 arrest. Our results demonstrate that 3D expression shifted the cells toward G0/G1 phase, and the extent of the arrest in G0/G1 phase was dependent on the dose of 3D plasmid that was transfected (0, 0.5, 1, 2 μg) (*R* = 0.897; *P* < 0.001). Similar results were observed for 3C (*R* = 0.924; *P* < 0.001) (Figures 8A,B). To further confirm the role of 3C and 3D in manipulating G0/G1 phase, we transfected PEGFP, 3C-PEGFP and 3D-PEGFP into 293T cells for analyzing successfully transfected cells, and it was found that in PEGFP-transfected cells the ratio of G0/G1 is 24.34 ± 1.89%, in 3C-PEGFP-transfected cells it is 70.79 ± 0.44% (*P* < 0.001, compared to PEGFP), and in 3D-PEGFP-transfected cells it is 70.79 ± 0.63% (*P* < 0.001, compared to PEGFP), meanwhile in three groups, for non-transfected cells the ratio of G0/G1 is 55.53 ± 1.07, 57.86 ± 0.24, and 58.60 ± 0.19%, respectively (Figures 8C,D). Therefore, these results suggest that the viral proteins of 3D and 3C may each contribute to the enhanced percentage of G0/G1 cells after CVA6 infection.

## Discussion

CVA6 is the primary causative agent for atypical HFMD, and epidemiological data indicates that outbreaks of CVA6-associated HFMD have markedly increased worldwide in recent years (Gao et al., 2016; Laga et al., 2016; Li J. S. et al., 2016; Li W. et al., 2016; Mirand et al., 2016). However, although the pathogenic mechanism of CVA6 has been partly studies (Zhang et al., 2017), the relationship between CVA6 and host cells was not very clear.

In the present study, the relationship between CVA6 and the host cell cycle was investigated, and our results demonstrate that CVA6 infection induces significant cell-cycle arrest in G0/G1 phase in the human rhabdomyosarcoma RD cell line. A number of viruses create a favorable environment for own replication through manipulating host cell-cycle (Chen and Makino, 2004; Helt and Harris, 2005; Dove et al., 2006; He et al., 2010; Yu et al., 2015). Therefore, our results are consistent with the possibility that the G0/G1-phase arrest induced by CVA6 might serve as a strategy for viral proliferation. To evaluate this possibility, cell cycle synchronization in G0/G1, S, and G2/M is executed by serum deprivation, thymidine and nocodazole treatment, respectively. Our results show that G0/G1 synchronization promotes CVA6 replication and viral production but not affecting virus entry, however that synchronization in S and G2/M phase decreases viral replication and inhibits viral production. These results suggest that G0/G1 phase is most favorable for CVA6 production, and that S or G2/M phase is inhibitory for viral production.

In our previous study, we determined that EV71 and CA16, which are the main agents leading to typical HFMD, induce cell cycle arrest in S phase to facilitate their own production (Yu et al., 2015), which is in contrast to the findings for CVA6 in this study. These results are surprising, given that EV71, CA16 and CVA6 each belong to the *Picornaviridae* family and cause HFMD. Although we do not know the reason for this difference, it could explain why EV71 and CVA6 have different characteristics, such as region of the epidemic (Europe and Asia), symptoms in clinic, and scope of the epidemic. We also demonstrated that human enterovirus 68 (EVD68), which is an alternate emerging pathogen that can cause severe respiratory disease, induces cell cycle arrest at G0/G1 phase (Wang et al., 2017). Therefore, CVA6 and EVD68 share this ability to regulate cell cycle progression at G0/G1. Similar to the results of our study, G2/M synchronization has been shown to inhibit the production of EV71 (Yu et al., 2015). Therefore, for treatment of either typical or atypical HFMD symptoms, agents inducing G2/M cell cycle arrest should be considered.

Cell cycle progression is controlled by the binding of CDKs with the corresponding cyclins. For example, cyclinD/CDK4 and cyclinD/CDK6 regulate the cell cycle in G0/G1 (Massagué, 2004), cyclin E/CDK2 regulates cellular S-phase entry from G1 (Hinds et al., 1992; Coverley et al., 2002; Yam et al., 2002), and cyclinB1/CDK1 regulates the mitotic process (Yu et al., 2013; Adeyemi and Pintel, 2014). To further understand the mechanism of G0/G1-phase arrest induced by CVA6 infection, host protein expression was examined including cyclinD1, CDK6, CDK4, cyclinE, CDK2, CDK1 and cyclinB1. Each of these proteins was down-regulated after CVA6 infection, though the timepoint of downregulation varied. Furthermore, the CyclinD1 inhibitory proteins P53, P21, and P16 were upregulated by CVA6 infection. These findings support a model in which: (1) CVA6 infection can arrest at G0/G1 phase by down-regulating CDK4, CDK6 and cyclinD1 and upregulating the P53-P21 and P16 pathway; (2) CVA6 infection can inhibit S phase entry by down-regulating cyclinE and CDK2 expression; (3) CVA6 infection can promote G2/M exit by down-regulating cyclinB1 and CDK1 expression.

Based on the well-characterized association between viral non-structural proteins and viral replication (Goh et al., 1998; Yuan et al., 2006; Yu et al., 2015), it was speculated that viral proteins responsible for CVA6 replication might manipulate the cell cycle progression. Our results prove that the expression of and 3D induces cell cycle arrest in G0/G1 phase, indicating that 3C (a protease) (Weng et al., 2009; Lei et al., 2012) and 3D (an RNA-dependent RNA polymerase) (Baltimore, 1964; Richards et al., 2006) are important to viral production. Therefore, our study demonstrates a new function for these proteins of CVA6 and implicates them as putative targets for the strategic development of new antiviral therapies.

## Author contributions

JY designed the experiments and wrote the paper. ZW, YW, JY, XM, FS, WH and SZ conducted the experiments. SW, JC, JL, BZ, YL, YZ and WZ prepared the virus, cell, plasmid and regents. JY, ZW, YW, XM, FS, WH, SZ, SW, JC, JL, BZ, YL, YZ and WZ analyzed, and discussed the data.

### Conflict of interest statement

The authors declare that the research was conducted in the absence of any commercial or financial relationships that could be construed as a potential conflict of interest.
